# Human T-Lymphotropic Virus type 1c subtype proviral loads, chronic lung disease and survival in a prospective cohort of Indigenous Australians

**DOI:** 10.1371/journal.pntd.0006281

**Published:** 2018-03-12

**Authors:** Lloyd Einsiedel, Hai Pham, Kim Wilson, Rebecca Walley, Jocelyn Turpin, Charles Bangham, Antoine Gessain, Richard J. Woodman

**Affiliations:** 1 Aboriginal Health Domain, Baker Heart and Diabetes Institute central Australia, Alice Springs Hospital, Alice Springs, Australia; 2 National Serology Reference Laboratory, Melbourne, Australia; 3 Flinders University/Northern Territory Rural Clinical School, Alice Springs Hospital, Alice Springs, Australia; 4 Section of Virology, Division of Infectious Diseases, Department of Medicine, Imperial College London, Norfolk Place, London, United Kingdom; 5 Institut Pasteur, Unité d’Epidémiologie et Physiopathologie des Virus Oncogènes, Département de Virologie, Paris, France, CNRS UMR 3569; 6 Flinders Centre for Epidemiology and Biostatistics, Flinders University, Adelaide, Australia; Center for Disease Control and Prevention, UNITED STATES

## Abstract

**Background:**

The Human T-Lymphotropic Virus type 1c subtype (HTLV-1c) is highly endemic to central Australia where the most frequent complication of HTLV-1 infection in Indigenous Australians is bronchiectasis. We carried out a prospective study to quantify the prognosis of HTLV-1c infection and chronic lung disease and the risk of death according to the HTLV-1c proviral load (pVL).

**Methodology/Principal findings:**

840 Indigenous adults (discharge diagnosis of bronchiectasis, 154) were recruited to a hospital-based prospective cohort. Baseline HTLV-1c pVL were determined and the results of chest computed tomography and clinical details reviewed. The odds of an association between HTLV-1 infection and bronchiectasis or bronchitis/bronchiolitis were calculated, and the impact of HTLV-1c pVL on the risk of death was measured.

Radiologically defined bronchiectasis and bronchitis/bronchiolitis were significantly more common among HTLV-1-infected subjects (adjusted odds ratio = 2.9; 95% CI, 2.0, 4.3). Median HTLV-1c pVL for subjects with airways inflammation was 16-fold higher than that of asymptomatic subjects. There were 151 deaths during 2,140 person-years of follow-up (maximum follow-up 8.13 years). Mortality rates were higher among subjects with HTLV-1c pVL ≥1000 copies per 10^5^ peripheral blood leukocytes (log-rank χ^2^ (2df) = 6.63, p = 0.036) compared to those with lower HTLV-1c pVL or uninfected subjects. Excess mortality was largely due to bronchiectasis-related deaths (adjusted HR 4.31; 95% CI, 1.78, 10.42 versus uninfected).

**Conclusion/Significance:**

Higher HTLV-1c pVL was strongly associated with radiologically defined airways inflammation and with death due to complications of bronchiectasis. An increased risk of death due to an HTLV-1 associated inflammatory disease has not been demonstrated previously. Our findings indicate that mortality associated with HTLV-1c infection may be higher than has been previously appreciated. Further prospective studies are needed to determine whether these results can be generalized to other HTLV-1 endemic areas.

## Author summary

The Human T-Lymphotropic Virus type 1 (HTLV-1) infects up to 20 million people worldwide who predominantly reside in resource-limited areas. The virus is associated with a haematological malignancy (adult T-cell leukaemia/lymphoma, ATL), and inflammatory diseases involving organ systems including the spinal cord, eyes and lungs. Determining the outcomes of infection in most HTLV-1 endemic areas is extremely difficult; however, the virus is highly endemic to central Australia where the Indigenous population has access to sophisticated medical facilities. We prospectively followed a large hospital-based cohort of Indigenous Australian adults that was well characterized with regard to base-line comorbid conditions, HTLV-1 serostatus and HTLV-1 proviral load (pVL). A higher baseline HTLV-1 pVL was strongly associated with an increased risk of airway inflammation (bronchitis/bronchiolitis and bronchiectasis) and death, which most often resulted from complications of bronchiectasis. Increased mortality due to an HTLV-1-associated inflammatory condition has not been demonstrated previously. The morbidity and mortality associated with HTLV-1 infection may therefore be substantially higher than has been assumed from an analysis of cohorts of subjects with adult T-cell leukaemia or HTLV-1-associated myelopathy. These findings have important implications for epidemiological research and for determining health care priorities in resource-limited settings.

## Introduction

The Human T-Lymphotropic Virus type 1 (HTLV-1) is an oncogenic retrovirus that preferentially infects CD4+ T cells[1]. Worldwide, HTLV-1 infects as many as 20 million people who predominantly dwell in areas of high endemicity in south-western Japan and developing countries of the Caribbean basin, South America and sub-Saharan Africa[2]. An endemic focus is present in central Australia[3] where more than 40% of Indigenous adults are HTLV-1c-infected in some remote communities[4].

Clinically significant sequelae of HTLV-1 infection include a haematological malignancy, Adult T cell Leukemia/Lymphoma (ATL), and inflammatory diseases, such as HTLV-1 associated myelopathy/tropical spastic paraparesis (HAM/TSP)[1]. In Japan and the Caribbean, life-time risks of HAM/TSP and ATL range between 0.3–4% and 1–5%, respectively[1]. Bronchiectasis is the most common clinical manifestation of HTLV-1 infection in Indigenous Australians, amongst whom the adult prevalence of this condition is the highest reported worldwide (>1%)[5,6]. Chest computed tomography has also revealed bronchiectasis in Japanese adults infected with HTLV-1; however, the most frequently reported radiological pattern of HTLV-1 associated pulmonary disease in this population is bronchitis/bronchiolitis[7,8], which has not been described in Indigenous Australians.

In endemic areas in Japan and Africa, HTLV-1 seropositivity is associated with increased mortality[9–12], which has been attributed to non-neoplastic conditions[9,10]. The interpretation of these studies is limited by their inability to control for clinically defined comorbid conditions that might independently increase mortality[9,11,12] [10]. For example, HTLV-1 seropositivity had no effect on mortality in a large hospital-based cohort of Indigenous Australian adults after adjusting for other medical conditions[13]. Given the close association between the number of HTLV-1-infected cells in peripheral blood (the HTLV-1 proviral load, pVL) and serious HTLV-1 associated complications[1,14], any influence of HTLV-1 infection on mortality might be revealed by stratifying outcomes according to HTLV-1 pVL. In central Australia, Indigenous adults with higher HTLV-1c pVL have more extensive, radiologically defined pulmonary injury[6] and are more likely to present with life-threatening bacterial infections[15]. A single, small study in Guinea-Bissau, where causes of death could not be ascertained, found that mortality increased with HTLV-1 pVL[16]. The present study was therefore commenced to quantify the prognosis of HTLV-1c infection and chronic lung disease and the risk of death according to the HTLV-1c pVL in a hospital-based cohort of Indigenous adults who were well characterized with regard to comorbid conditions and for whom causes of death could be accurately determined in nearly all cases.

## Methods

### Study setting

Alice Springs Hospital (ASH) is the only medical facility serving central Australia, an area of >1,000,000 km^2^. Critically ill patients are transferred by air to ASH, which has sophisticated diagnostic capabilities.

### Recruitment

**Fig 1:**
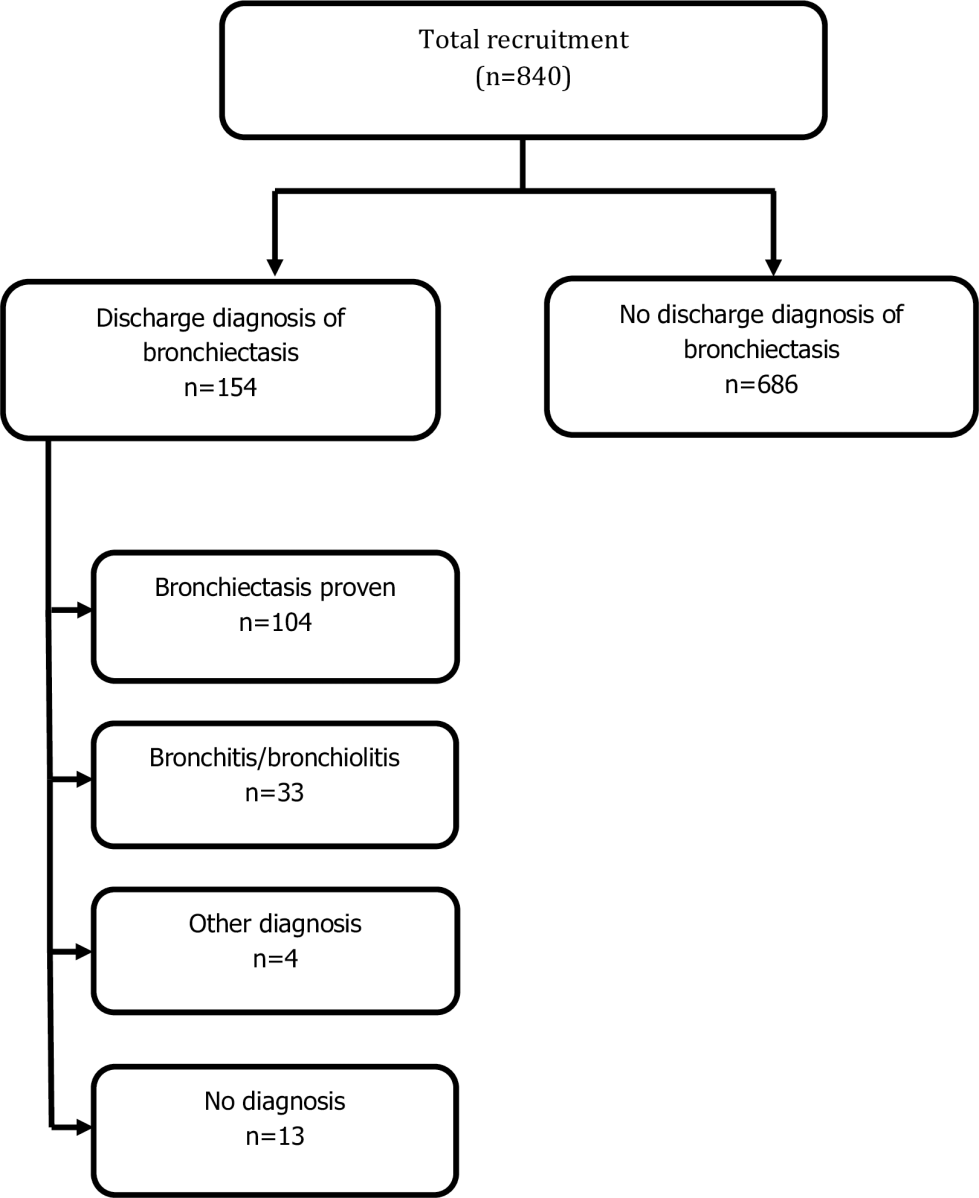
Recruitment based on discharge diagnosis. Subjects with a discharge diagnosis of bronchiectasis were examined by chest high-resolution computed tomography, which confirmed the diagnosis in 104 cases and revealed bronchitis/bronchiolitis in 33 cases (bronchitis alone, 20; bronchitis/bronchiolitis, 12; bronchiolitis alone, 1). Other diagnoses included emphysema (3) and pulmonary embolus (1). No cause of chronic cough could be found in 13 cases.

All Indigenous patients aged >15 years with a discharge diagnosis of bronchiectasis, 1^st^ June 2008 to 31^st^ December 2013, were identified from the ASH patient management database, which coordinates all in-patient and out-patient hospital activities. Indigenous status was determined from self-reported data obtained at admission, as recorded in the patient information database. Potential subjects were offered enrolment when next admitted for >48 hours. Among 165 eligible cases, 154 were recruited (eleven subjects left hospital before recruitment was possible). Written reports for chest high-resolution computed tomography (cHRCT) were reviewed for all subjects, confirming bronchiectasis in 104 cases and bronchitis with or without bronchiolitis in 33 cases (bronchitis alone, 20; bronchitis and bronchiolitis, 12; bronchiolitis alone, 1)(Fig 1). Patients with chronic pulmonary disease were treated according to local guidelines which includes antibiotic therapy for infective exacerbations [17]. A further 686 Indigenous patients aged >15 years who were admitted for >48 hours were prospectively recruited during the same period. These control subjects had no evidence of lower respiratory tract infection at the time of recruitment, no recorded discharge diagnosis of bronchiectasis, and no clinical or radiological evidence of bronchiectasis, Research team members who were unaware of HTLV-1 serostatus were responsible for recruitment (Fig 1).

Demographic and clinical details were extracted from medical records at the time of recruitment using a standardized data-collection form. HTLV-1 associated conditions were identified from medical records at baseline and study end. No control patient developed chronic pulmonary disease during the study period. Mortality data was obtained at study end from the ASH patient management database, and the cause of death was determined from death certificates held in Registries in the Northern Territory of Australia and South Australia. Death certificates were not available for four subjects who died in remote communities in Western Australia, for whom a cause of death was sought from the responsible remote clinic.

### Definitions

Bronchitis was diagnosed where cHRCT revealed bronchial wall thickening or dilatation not fulfilling criteria for bronchiectasis, and bronchiolitis where cHRCT revealed multiple centrilobular nodules or a ‘tree-in-bud’ pattern[8]. Chronic obstructive pulmonary disease (COPD) required a clinical diagnosis in the medical record and appropriate chest X-ray findings. Emphysema without bronchial wall injury or bronchiolitis was recorded for 18 subjects with COPD examined by cHRCT. Chest HRCT was not performed on 12 subjects who did not meet criteria for such imaging[17]. No subject with symptoms consistent with HAM/TSP received lumbar puncture; the diagnosis was therefore considered ‘probable’ in all cases. Asymptomatic HTLV-1-infected subjects were those without radiological evidence of airway inflammation or recognized HTLV-1 associated conditions [1]. Residence >80 km from the township of Alice Springs was defined as remote.

### Ethics approval

The study was approved by the Central Australian Human Research Ethics Committee. All patients, and their parents/guardians if aged <18 years, gave written informed consent in primary languages.

### HTLV-1 serologic and molecular studies

Whole blood samples were collected from each participant at the time of recruitment. Peripheral blood buffy coats (PBBC) were prepared, and plasma and PBBCs were stored at ASH at -80° C until transfer to the National Serology Reference Laboratory, Melbourne. Samples were screened for antibodies to HTLV-1 using both an enzyme immunoassay (Murex HTLV-I + II, DiaSorin, Italy) and a particle agglutination assay (Serodia HTLV-1, Fujirebio, Tokyo, Japan). Any sample reactive on either screening assay was tested by Western blot (HTLV-I/II Blot2.4, MP Biomedicals Asia Pacific Pte. Ltd., Singapore) and HTLV-1c PCR. Primers and fluorescently labelled hydrolysis probes were designed to target a highly conserved 88 bp fragment of the *gag* gene in the p19 coding region of the Australo-Melanesian HTLV-1 subtype C[18] and multiplexed with primers and probes to the albumin gene[19]. SP cells were used to generate a standard curve from which HTLV-1 pVL (copies per 10^5^ peripheral blood leukocytes; PBL) was calculated. Samples and standards were extracted using the Qiagen QIA blood Mini Extraction kit and the extracts amplified on a Stratagene Mx3000p Real Time PCR Instrument (Integrated Sciences). The extract (5 μL) was added to 20 μL of Master mix containing 2 x Brilliant Multiplex QPCR Master Mix (Agilent Technologies) 0.3 μM of each primer (Gene works) and 0.16 μM of each probe (Sigma-Aldrich) and amplified at 95°C for 10 minutes, 45 cycles at 95°C for 30 seconds, 65°C for 60 seconds and 72°C for 60 seconds.

### Oligoclonality

The clonality of HTLV-1-infected PBLs was determined by high-throughput sequencing of PBBC cell genomic DNA. The oligoclonality index (OCI) was calculated as previously described[20], and then adjusted to limit underestimation of the OCI due to the small observed number of proviruses[21]. The OCI provides a measure of the non-uniformity of the clone abundance distribution of the infected cell population: OCI = 1 indicates perfect monoclonality (only one clone constitutes the total proviral load); OCI = 0 indicates perfect polyclonality (all clones have the same abundance)[20]. Samples were selected for clonality analysis if subjects had HTLV-1 pVL >100 copies per 10^5^ PBL, were HBsAg negative and strongyloides seronegative. Although 53 subjects met these criteria, technical difficulties prevented analysis for nine subjects (inadequate number of unique integration sites to accurately determine OCI, 7; unable to sequence integration site, 2). The OCI was therefore compared between 29 asymptomatic and 15 symptomatic subjects (bronchiectasis, 10; bronchitis/bronchiolitis, 3; uveitis, 2).

### Statistical analysis

All analysis was performed using Stata version 14.2 (StataCorp, College Station, USA). HTLV-1 pVL was log-transformed and also categorized as low if <1000 and high if ≥1000 per 10^5^ PBL, a cut-off that has been associated with an increased risk of HAM/TSP[22]. Differences between subjects who were HTLV-1 uninfected, those with low HTLV-1 pVL, and those with high HTLV-1 pVL were assessed using ANOVA for continuous variables and chi-squared tests for categorical variables. For statistical purposes, causes of death were grouped into six non-overlapping categories: bronchiectasis, sepsis, cardiovascular disease, malignancy, chronic kidney disease and chronic liver disease. We used survival analysis to determine the association between HTLV-1 pVL and both overall and cause-specific mortality. Subjects were followed until either date of death or 30^th^ March 2015. The association between overall mortality and HTLV-1 pVL was assessed using log-rank tests and Kaplan-Meier curves in univariate analysis and using Cox regression for multivariate analysis. Associations with cause-specific mortality were assessed using competing risks analysis with all causes except the specific cause of interest treated as a competing risk. Where HTLV-1 pVL was treated as a categorical variable we also tested for a trend by creating a continuous variable with value zero for those uninfected, and with the median value of HTLV-1 pVL for those in the low and high pVL categories. Predictors of chronic airways inflammation were assessed using multivariate binary logistic regression. A 2-sided Type 1 error rate of p<0.05 was regarded as indicating statistical significance in each analysis.

## Results

Demographic and clinical characteristics are presented in Table 1. Among 840 subjects recruited to the study, 307 (36.5%) were HTLV-1 infected (HTLV-1 Western blot positive, 268; Western blot indeterminate/HTLV-1c PCR positive, 39).

**Table 1:** Demographics and clinical characteristics of indigenous adults according to HTLV-1 status and HTLV-1 proviral load.

	No HTLV-1 (n = 533)	Low HTLV-1 proviral load (n = 212)	High HTLV-1 proviral load (n = 95)	p-value^1^
Age at test, years (mean±SD)	46.2±15.3	51.6±13.7	51.1±13.8	<0.001
Male, n (%)	305 (57.2)	119 (56.1)	58 (61.0)	0.72
Adult residency, n (%)^a^ Urban Remote Town Camp Other States	75 (14.1) 376 (70.5) 81 (15.2) 1 (0.2)	25 (11.8) 138 (65.1) 48 (22.6) 1 (0.5)	11 (11.6) 64 (67.4) 20 (21.1) 0 (0.0)	0.20
Bronchiectasis, n (%)^b^	45 (8.4)	35 (16.5)	24 (25.3)	<0.001
Bronchitis/Bronchiolitis, n (%)^c^	12 (2.3)	11 (5.2)	10 (10.5)	0.001
COPD, n (%)^d^	16 (3.0)	11 (5.2)	3 (3.2)	0.34
CHF, n (%)	53 (9.9)	22 (10.4)	8 (8.4)	0.91
IHD, n (%)	103 (19.3)	43 (20.3)	16 (16.8)	0.79
Diabetes, n (%)	274 (51.4)	114 (53.8)	51 (53.7)	0.81
ESKD, n (%)	63 (11.9)	30 (14.3)	10 (10.5)	0.60
CLD, n (%)	53 (9.9)	29 (13.7)	12 (12.6)	0.28
Malignancy, n (%)	19 (3.6)	9 (4.2)	5 (5.3)	0.62
Alcohol, n (%)^e^	337 (63.2)	122 (57.6)	48 (50.5)	0.04
Strongyloides serology^f^	111/471 (23.5)	51/180 (28.3)	18/70 (25.7)	0.08
Deaths, n(%)	85 (15.9)	43 (20.2)	27 (28.4)	
Age at death, years (mean±SD)	53.6±13.5	54.8±9.2	51.4±13.4	0.54

a, residence in adulthood. Urban, residence in Alice Springs; Remote, residence in remote community ≥80 km from Alice Springs; Town camp, residence in town camp or community <80 km from Alice Springs; other states, resident outside central Australia

b, chest HRCT proven bronchiectasis

c, chest HRCT findings consistent with bronchitis or bronciolitis

d, clinical and radiological evidence of chronic obstructive pulmonary disease

e, history of harmful alcohol consumption documented in medical records or any hospital admission with alcohol related complications

f, Strongyloides serology was performed for 520, 206 and 88 subjects who were HTLV-1 negative, HTLV-1 infected with low HTLV-1 proviral load and high HTLV-1 proviral load, respectively. Data for 49, 26 and 18 subjects who recorded equivocal serological results were excluded from the analysis.

^1^ Difference between the three groups using ANOVA for continuous variables and Fishers Exact test for categorical variables; Abbreviations: COPD, Chronic obstructive pulmonary disease; CHF, Congestive heart failure; IHD, Ischaemic heart disease; ESKD, end-stage kidney disease; CLD, chronic liver disease.

### Clinical associations

Radiologically defined airways inflammation was more common among HTLV-1c-infected subjects (Table 1). Bronchiectasis was confirmed in 59/307 (19.2%) HTLV-1c-infected subjects and 45/533 (8.4%) HTLV-1c uninfected subjects. Similarly, cHRCT revealed bronchitis/bronchiolitis in 21/307 (6.8%) HTLV-1c-infected subjects and 12/533 (2.3%) who were HTLV-1c uninfected (Table 1).

**Fig 2:**
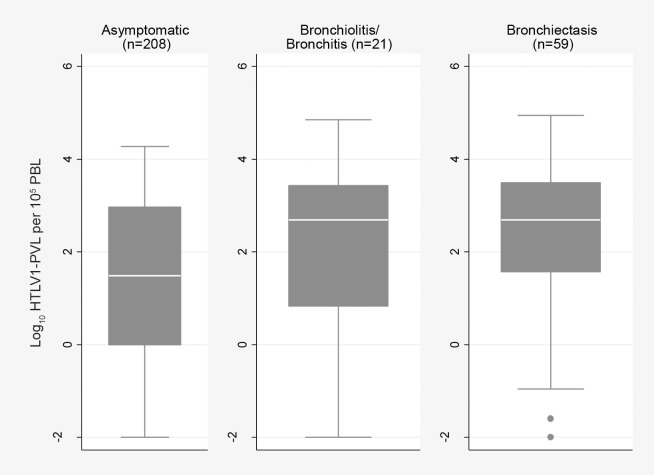
HTLV-1c proviral load compared between asymptomatic subjects, bronchiolitis/bronchitis and bronchiectasis. Subjects with chronic airways disease were examined by chest high resolution computed tomography. Median (IQR) HTLV-1c pVL for subjects with bronchiolitis/bronchitis (2.69 (0.82, 3.43) log_10_ copies per 10^5^ PBL) and bronchiectasis (2.69 (1.57, 3.49) log_10_ copies per 10^5^ PBL) were significantly higher than that of the asymptomatic group (1.48 (-0.001, 2.97) log_10_ copies per 10^5^ PBL)(asymptomatic vs bronchiectasis, p = 0.001; asymptomatic vs bronchitis/bronchiolitis, p = 0.042). Asymptomatic subjects exclude: i) those with other HTLV-1 associated conditions (infective dermatitis, 2; probable HAM/TSP, 2; uveitis, 2; crusted scabies, 4; ATL; 1), ii) five subjects with a discharge diagnosis of bronchiectasis without radiological evidence of bronchiectasis, bronchitis or bronchiolitis and iii) three subjects who presented with neurological symptoms for whom HAM/TSP could not be excluded due to cognitive impairment.

Compared to the median HTLV-1c pVL of asymptomatic subjects (n = 208, 30.4 copies per 10^5^ PBL (min, 0.01; max, 18600), those for subjects with bronchiectasis (n = 59, 494 copies per 10^5^ PBL; min, 0.01; max, 87900) (p = 0.001) and bronchitis/bronchiolitis (n = 21, 486 copies per 10^5^ PBL; min, 0.01; max, 70200)(p = 0.042) were 16-fold higher,(Fig 2). Median HTLV-1c pVL of subjects with any airways inflammation (bronchiectasis and bronchitis/bronchiolitis; 490 copies per 10^5^ PBL; min, 0.01; max 87900) was 16-fold higher than that of asymptomatic subjects (p<0.001). Few other recognised causes of bronchiectasis were found (Table 2).

**Table 2:** Investigations for causes of radiologically defined airways inflammation according to HTLV-1 serostatus.

	AFB	ANA	ASP	Ig Deficiency	IgG subclass Deficiency	A1AT Deficiency
HTLV-1 Uninfected	0/39	2/48^a^	0/47	1/48^b^	1/44^b^	0/42
HTLV-1 Infected	1/60^c^	1/59^d^	0/53	0/53	0/49	0/50

Denominator gives number of subjects who received each test.

a, bronchitis/bronchiolitis associated with Sjogren’s syndrome (1), rheumatoid arthritis (1)

b, bronchiectasis associated with Immunoglobulin A and G_1–3_ deficiency

c, *Mycobacterium avium/intracellulare* complex repeatedly cultured from sputum in one case with bronchitis/bronchiolitis (HTLV-1c pVL, 9.4 copies per 10^5^ PBL)

d, Antinuclear antibody positive but not deemed to be significant for a 32 year old, HTLV-1c infected man who died with rapidly progressive haemophagocytic lymphohistiocytosis (HTLV-1c pVL, 1951 copies per 10^5^ PBL). Abbreviations: AFB, ≥3 adequate sputum cultures for mycobacteria or any case in which a mycobacterium was isolated; ANA, anti-nuclear antibodies; A1AT deficiency: deficiency of alpha 1 anti-trypsin; ASP, aspergillus precipitins; Ig, immunoblobulin; Ig Deficiency, levels of Immunoglobulin classes A or G less than lower limit of normal; IgG subclass deficiency, levels of IgG subclasses less than lower limit of normal.

In a multivariate model that controlled for demographic factors, smoking and harmful alcohol consumption, HTLV-1 infection increased the risk of any airways inflammation 2.9-fold (p<0.001) (Table 3), while HTLV-1c pVL ≥1000 copies per 10^5^ PBL increased the risk 2.2-fold among HTLV-1-infected subjects (p = 0.006) (Table 4).

**Table 3:** Multivariable predictors of radiologically defined airway inflammation^a^ among all subjects^1^ (n = 840)^b^.

	Model 1		Model 2		Model 3	
	OR (95% CI)	p-value^1^	OR (95% CI)	p-value^1^	OR (95% CI)	p-value^1^
HTLV-1 Infected	2.9 (2.0, 4.3)	<0.001	2.8 (1.9, 4.2)	<0.001	2.9 (2.0, 4.3)	<0.001
Age^c^			1.0 (0.99, 1.02)	0.28	1.0 (0.99, 1.02)	0.29
Gender			1.11 (0.8, 1.6)	0.60	1.2 (0.78, 1.80)	0.43
Residence^d^ Urban					1.00	-
Remote					0.8 (0.4, 1.4)	0.39
Town Camp Other States					1.1 (0.6, 2.1) 3.7 (0.2, 64.7)	0.77 0.38
Ever smoked					0.8 (0.5, 1.2)	0.30
Alcohol^e^					1.5 (0.9, 2.2)	0.09

a, bronchiectasis, bronchitis or bronchiolitis identified by chest high resolution computed tomography

b, HTLV-1 seronegative, 533; HTLV-1 infected, 307 (Western blot positive, 268; HTLV-1 Western blot indeterminate/HTLV-1 PCR positive, 39)

c, per 1 year increase in age

d, Residence in adulthood

e, history of harmful alcohol consumption documented in medical records or any hospital admission with alcohol related complications. Urban, residence in Alice Springs; Remote, residence in remote community ≥80 km from Alice Springs; Town camp, residence in town camp or community <80 km from Alice Springs; other states, resident outside central Australia.

^1^ Using multivariate binary logistic regression. Model 1: unadjusted, Model 2: adjusted for age and gender, Model 3: additionally adjusted for area of residence, previous smoking history and alcohol abuse. p-value based on the Wald test of the associated odds ratio beta coefficient.

**Table 4:** Multivariable predictors of bronchiectasis^a^ among HTLV-1 infected subjects^1^ (n = 307).

	Model 1		Model 2		Model 3	
	OR (95% CI)	p-value	OR (95% CI)	p-value	OR (95% CI)	p-value
pVL Low^b^ pVL High^c^	1.00 2.0 (1.2, 3.4)	0.01	1.00 2.0 (1.2, 3.4)	0.01	1.0 2.2 (1.2, 3.7)	0.006
Age^d^			1.0 (0.98, 1.01)	0.61	1.0 (0.98, 1.01)	0.63
Gender			1.4 (0.8, 2.3)	0.25	1.4 (0.8, 2.6)	0.22
Residence^e^ Urban					1.00	-
Remote					1.1 (0.5, 2.6)	0.84
Town Camp					1.7 (0.6, 4.4)	0.29
Ever smoked					0.8 (0.4, 1.5)	0.47
Alcohol^f^					1.6 (0.9, 2.9)	0.10

a, diagnosed by chest high resolution computed tomography

b, pVL low, HTLV-1 proviral load<1000 copies per 10^5^ PBL

c, pVL high, HTLV-1c proviral load ≥ 1000 copies per 10^5^ PBL

d, per 1 year increase in age

e, Residence in adulthood

f, history of harmful alcohol consumption documented in medical records or any hospital admission with alcohol related complications. Urban, residence in Alice Springs; Remote, residence in remote community ≥80 km from Alice Springs; Town camp, residence in town camp or community <80 km from Alice Springs; other states, resident outside central Australia.

^1^ Using multivariate binary logistic regression. Model 1: unadjusted, Model 2: adjusted for age and gender, Model 3: additionally adjusted for area of residence, previous smoking history and alcohol abuse. p-value based on the Wald test of the associated odds ratio beta coefficient.

Other HTLV-1 associated conditions included infective dermatitis (4), crusted scabies (4), probable HAM/TSP (2) and uveitis (2). Four subjects with HTLV-1-associated bronchiectasis had other sequelae of HTLV-1 infection (infective dermatitis, 2; HAM/TSP, 1; uveitis, 1). There was no difference in strongyloides seropositivity between groups (Table 1), nor was there any difference in log-transformed HTLV-1c pVL using linear regression (p = 0.409); median (IQR) HTLV-1c pVL copies per 10^5^ PBL for subjects who were strongyloides seronegative (133; IQR 2.1, 1387), seropositive (61; IQR 1, 1039) and those with equivocal strongyloides serological results (190; IQR 2.7, 2473).

Although the risk of malignancy was not increased among HTLV-1c infected subjects, one subject (HTLV-1c pVL, 5500 copies per 10^5^ PBL) developed ATL during follow-up, one developed penile cancer (HTLV-1c pVL, 70200 copies per 10^5^ PBL) and another metastatic anal cancer (HTLV-1c pVL, 28000 copies per 10^5^ PBL).

### Clonality

The median OCI did not differ between asymptomatic (0.401; IQR 0.350, 0.485) and symptomatic groups (0.411; IQR 0.355, 0.552) (p = 0.51) or when symptomatic subjects with uveitis were excluded from the analysis (symptomatic group median OCI 0.429; IQR 0.369, 0.552) (p = 0.341). Median log_10_ HTLV-1c pVL copies per 10^5^ PBL in subjects selected for clonality analysis did not differ between asymptomatic subjects (0.947; IQR 0.342, 1.964) and those with airways inflammation (1.50; IQR 0.669, 4.074)(p = 0.20).

### Mortality

During 2140 person-years of follow-up, 155 deaths were recorded (HTLV-1 uninfected, 85; HTLV-1 infected, 70). Non-bronchiectasis causes of death were infections (37)(lower respiratory tract infections, 15), cardiovascular disease (37), malignancy (15), end-stage kidney disease (ESKD) (15), chronic liver disease (10), intracerebral haemorrhage (4), primary pulmonary hypertension (2) and amyloidosis (1).

**Fig 3:**
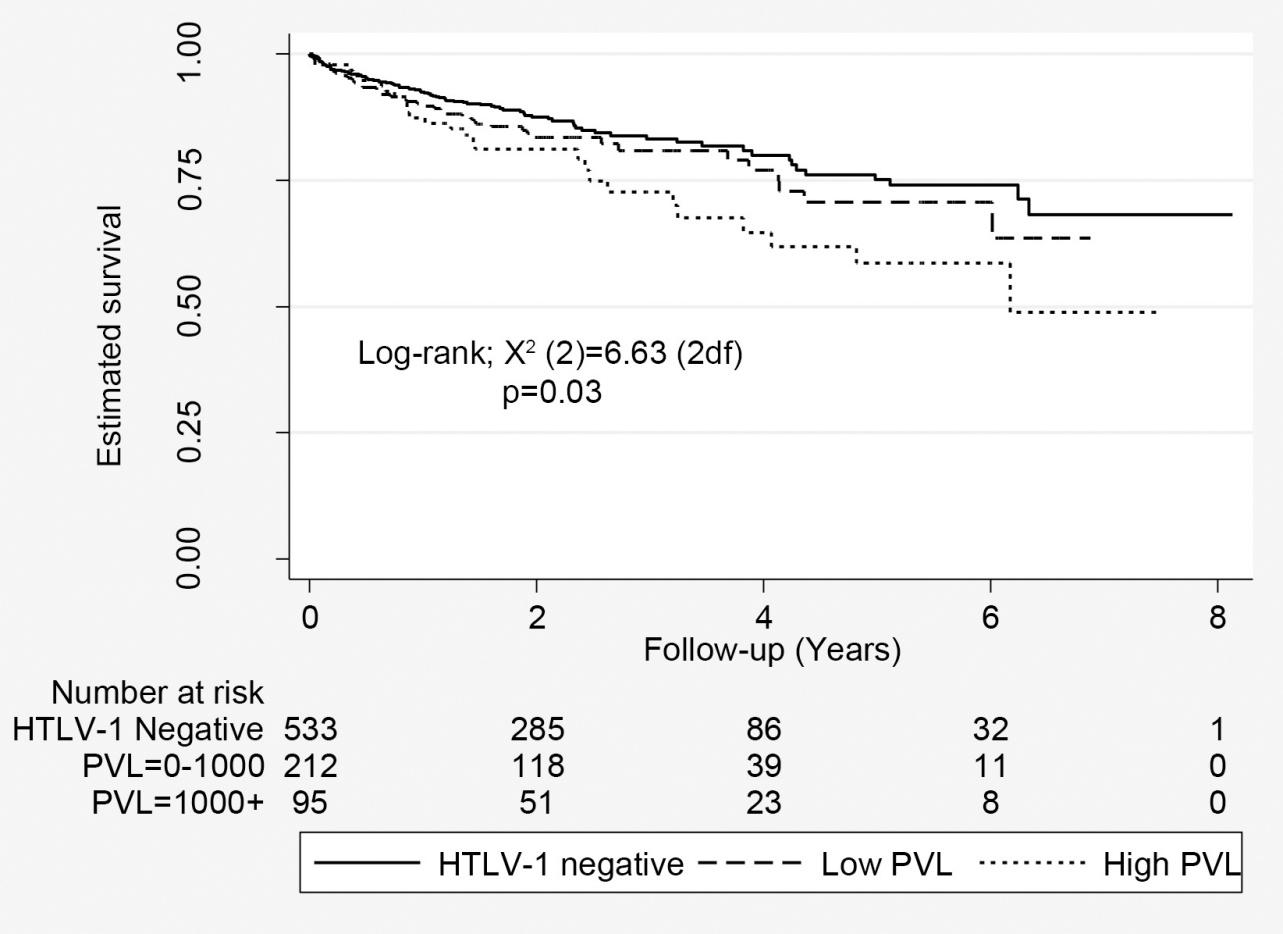
Estimated survival according to HTLV-1 proviral load. Includes 533 HTLV-1 uninfected subjects, 212 subjects with low HTLV-1c proviral load (<1000 copies per 10^5^ peripheral blood leukocytes) and 95 subjects with high HTLV-1c proviral load (≥1000 copies per 10^5^ peripheral blood leukocytes).

Subjects with high HTLV-1c pVL were more likely to die during the study period (Fig 3). Mortality rates for high HTLV-1c pVL, low HTLV-1c pVL and HTLV-1 uninfected subjects were 28.4% (27/95), 20.2% (43/212) and 15.9% (85/533), respectively (p = 0.011). The unadjusted HR for death among subjects with low and high HTLV-1c pVL were 1.24 (95% CI, 0.85, 1.81) and 1.75 (95% CI, 1.13, 2.670), respectively (p = 0.021 for trend). The statistical significance was diminished after adjusting for age, gender, place of residence and harmful alcohol consumption (p = 0.084). The effect of HTLV-1c pVL on overall mortality was lost in a multivariate model that included bronchiectasis (aHR, 1.045; 95% CI, 0.658–1.660) (Table 5). Other predictors of death were age at test, male gender and comorbid conditions (Table 5).

**Table 5:** Multivariate predictors of death among 840 Indigenous adults.

	HR	95% CI	p-value^1^
Age at test^a^	1.025	1.011–1.038	<0.001
Male gender	1.848	1.275–2.677	0.001
Adult residence^b^, Urban Remote Town Camp Other	reference 0.832 1.432 1.286	0.492–1.407 0.810–2.534 0.158–10.438	0.493 0.217 0.815
No HTLV-1 HTLV-1 pVL low^c^ HTLV-1 pVL high^d^	1.000 0.843 1.040	- 0.571–1.245 0.657–1.650	- 0.391 0.865
Bronchiectasis^e^	3.534	2.293–5.445	<0.001
COPD^f^	0.761	0.476–1.218	0.256
CHF	3.289	2.089–5.178	<0.000
IHD	1.694	1.150–2.494	0.008
Diabetes	0.972	0.658–1.434	0.885
ESKD	2.049	1.317–3.190	0.001
CLD	2.608	1.792–3.793	<0.001
Malignancy	4.101	2.472–6.803	<0.001
Alcohol^g^	0.702	0.488–1.008	0.055

a, per 1 year increase in age at test

b, Residence in adulthood. Urban, residence in Alice Springs; Remote, residence in a community ≥80 km from Alice Springs; Town Camp, residence in town camp or community <80 km from Alice Springs

c, HTLV-1 proviral load <1000 copies per 10^5^ peripheral blood leukocytes

d, HTLV-1 proviral load ≥ 1000 copies per 10^5^ peripheral blood leukocytes

e, chest HRCT proven bronchiectasis

f, clinical and radiological evidence of chronic obstructive pulmonary disease

g, history of harmful alcohol consumption documented in medical records or any hospital admission with alcohol related complications. Abbreviations: COPD, Chronic obstructive pulmonary disease; CHF, Congestive heart failure; IHD, Ischaemic heart disease; ESKD, end-stage kidney disease; CLD, chronic liver disease.

^1^p-value based on the Wald test of the associated hazard ratio beta coefficient.

#### Cause-specific mortality

Relative to HTLV-1c uninfected subjects, unadjusted hazard ratios for a bronchiectasis-related death were 4.73 (95% CI, 2.05, 10.9) and 1.44 (95% CI, 1.02, 6.49) for subjects with high and low HTLV-1c pVL, respectively (Table 6).

**Table 6:** Hazard ratios for all-cause and cause-specific mortality according to HTLV-1 proviral load status.

		HTLV-1 Negative N = 533 (63.5%)	HTLV-1 pVL Low N = 212 (25.2%)	HTLV-1 pVL High N = 95 (11.3%)	P-value for trend^1^
All-cause mortality	Events, n (%)	83 (55.0)	41 (27.1)	27 (17.9)	
	Unadjusted	1.00	1.24 (0.85, 1.81)	1.75 (1.13, 2.70)	0.02
	Multivariate^2^	1.00	1.00 (0.69, 1.47)	1.43 (0.92, 2.23)	0.09
Cause-specific mortality^3^					
*Bronchiectasis*	Events, n (%)	12 (41.4)	7 (24.1)	10 (34.5)	
	Unadjusted	1.00	1.44 (1.02, 6.49)	4.73 (2.05, 10.94)	<0.001
	Multivariate^2^	1.00	1.23 (0.69, 4.58)	4.31 (1.78, 10.42)	0.001
*CVD*	Events, n (%)	25 (67.6)	8 (21.6)	4 (10.8)	
	Unadjusted	1.00	0.78 (0.35, 1.74)	0.81 (0.29, 2.30)	0.78
	Multivariate^2^	1.00	0.66 (0.30, 1.46)	0.66 (0.24, 1.84)	0.57
*Liver Disease*	Events, n (%)	4 (40.0)	5 (50.0)	1 (10.0)	
	Unadjusted	1.00	3.10 (0.83, 11.6)	1.38 (0.16, 11.6)	0.89
	Multivariate^2^	1.00	2.84 (0.75, 10.8)	1.30 (0.16, 10.8)	0.81
*Malignancy*	Events, n (%)	9 (60.0)	4 (26.7)	2 (13.3)	
	Unadjusted	1.00	1.08 (0.34, 3.43)	1.05 (0.23, 4.70)	0.98
	Multivariate^2^	1.00	0.74 (0.20, 2.83)	0.74 (0.16, 3.44)	0.79
*Other*	Events, n (%)	6 (60.0)	3 (30.0)	1 (10.0)	
	Unadjusted	1.00	1.23 (0.31, 4.92)	0.77 (0.10, 5.95)	0.75
	Multivariate^2^	1.00	1.13 (0.27, 4.7)	0.71 (0.09, 5.41)	0.71
*Renal Disease*	Events, n (%)	11 (68.8)	4 (25.0)	1 (6.2)	
	Unadjusted	1.00	0.88 (0.28, 2.75)	0.45 (0.06, 3.56)	0.46
	Multivariate^2^	1.00	0.74 (0.24, 2.30)	0.39 (0.05, 3.04)	0.42
*Sepsis*	Events, n (%)	19 (51.3)	10 (27.0)	8 (21.6)	
	Unadjusted	1.00	1.26 (0.58, 2.70)	2.17 (0.95, 4.96)	0.08
	Multivariate^2^	1.00	1.07 (0.50, 2.30)	1.81 (0.77, 4.29)	0.16

Hazard ratios were assessed using Cox-regression for all-cause mortality and competing risks regression for cause-specific mortality.

^1^ p-value for test of HTLV-1 pVL trend across the 3 categories conducted using the median pVL value for each category.

^2^Adjusting for alcohol, place of adult residence (Alice Springs, Remote, Town camp or other States), age at test and gender.

^3^All other causes of death were treated as a competing risk. HTLV-1c pVL low, proviral load <1000 copies per 10^5^ peripheral blood leukocytes; HTLV-1c pVL high, proviral load ≥ 1000 copies per 10^5^ peripheral blood leukocytes. Abbreviations: CVD, cardiovascular disease. Cause of death not determined for four subjects

**Fig 4:**
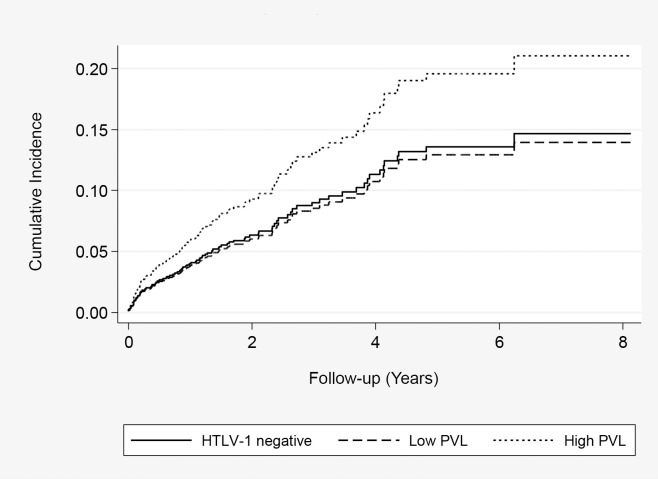
Cumulative death rate due to bronchiectasis. Estimated using competing-risks Cox regression among 533 HTLV-1 uninfected subjects, 212 subjects with low HTLV-1 proviral load (<1000 copies/10^5^ peripheral blood leukocytes) and 95 with high HTLV-1c proviral load (≥1000 copies/10^5^ peripheral blood leukocytes).

The effect of HTLV-1c pVL on bronchiectasis-related mortality remained significant for subjects with high HTLV-1c pVL (4.31; 95% CI, 1.78, 10.4) when other causes of death were treated as competing risks and after adjusting for alcohol, place of residence, age at test, and gender (Table 6) (Fig 4). The risk of a bronchiectasis-related death increased 4.5% with each 100 HTLV-1c copies per 10^5^ PBL when median values for proviral load were treated as a continuous variable in an adjusted model (p = 0.009 for trend). This effect was not apparent among the 736 subjects without bronchiectasis (Table 7). Causes of bronchiectasis-related deaths included lower respiratory tract infections (15), respiratory failure (13) and haemoptysis (1).

**Table 7:** Effect of HTLV-1c pVL on all-cause mortality according to bronchiectasis status.

		HTLV-1 Negative	HTLV-1 pVL Low	HTLV-1 pVL High	P-value for trend^1^
No Bronchiectasis		N = 488 (66.3%)	N = 177 (24.0%)	N = 71 (9.7%)	
	Events, n (%)	69 (59.5)	34 (29.3)	13 (11.2)	
	Unadjusted	1.00	1.34 (0.89, 2.02)	1.21 (0.67, 2.19)	0.72
	Multivariate^2^	1.00	1.10 (0.72, 1.67)	0.89 (0.49, 1.63)	0.63
Bronchiectasis		N = 45 (43.2%)	N = 35 (33.7%)	N = 24 (23.1%)	
	Events, n (%)	14 (40.0)	7 (20.0)	14 (40.0)	
	Unadjusted	1.00	0.62 (0.25, 1.54)	1.75 (0.83, 3.72)	0.03
	Multivariate^2^	1.00	0.50 (0.20, 1.29)	2.04 (0.89, 4.69)	0.009

Hazard ratios were assessed using Cox-regression for all-cause mortality and competing risks regression for cause-specific mortality.

^1^p-value for test of HTLV-1 pVL trend across the 3 categories conducted using the median pVL value for each category.

^2^Adjusting for alcohol, place of adult residence (Alice Springs, Remote, Town camp or other States), age at test and gender. All other causes of death were treated as a competing risk.

Mean (±SD) age at death for bronchiectasis-related deaths (49.5±15.3 years) was lower than that of subjects who died due to cardiovascular disease (55.0±11.5 years)(p = 0.073), sepsis (56.7±16.7)(p = 0.044) or malignancy (60.1±9.3) (p = 0.007).

## Discussion

In a large hospital-based cohort of Indigenous Australian adults, a higher baseline HTLV-1c pVL prospectively predicted a bronchiectasis-related death, which occurred at a mean age of only 49.5 years. In addition to confirming a previously reported association between HTLV-1c infection and bronchiectasis[5], the present study also revealed an association with bronchitis/bronchiolitis. Airways inflammation was strongly associated with higher HTLV-1c pVL[6,15]. The median HTLV-1c pVL of subjects with radiologically defined airways inflammation was 16-fold higher than that for asymptomatic HTLV-1-infected subjects, and risk of airway inflammation increased three-fold among subjects with higher HTLV-1c pVL in an adjusted model.

HTLV-1 associated inflammatory diseases are thought to result from a genetically determined, inefficient cytotoxic T lymphocyte response, permitting widespread dissemination of the virus in a large number of HTLV-1-infected T-cell clones, which is reflected in a high HTLV-1 pVL[23]. Organ infiltration by HTLV-1-infected lymphocytes then leads to high local HTLV-1 antigen levels, provoking an immune response and tissue injury following the release of pro-inflammatory cytokines and chemokines[23]. Although this has been best studied for the prototypical HTLV-1 associated disease, HAM/TSP, HTLV-1 infection is also associated with inflammation in other organs[14] including the lungs [24]. Consistent with the presumed mechanism of pathogenesis of HAM/TSP [23], pulmonary involvement is associated with infiltration of HTLV-1-infected lymphocytes[25,26], increased *tax/rex* mRNA[27] expression, and an inflammatory cytokine milieu in bronchoalveolar lavage fluid[27]. In large Japanese case series, cHRCT was abnormal in 30–61% of HTLV-1 infected subjects of whom 23.6–29.5% had a bronchitis/bronchiolitis pattern of disease and 15.6–22.5% had frank bronchiectasis[7,8]. The pathological correlate of these observations is lymphocyte infiltration in bronchiole walls[7]. Persistent HTLV-1-mediated airways inflammation may therefore lead to progressive bronchial wall dilatation, and bronchiectasis. High rates of bronchiectasis among HTLV-1-infected Japanese adults[7,8], and associations between HAM/TSP and bronchiectasis in UK [28] and Brazilian cohorts [29] suggest that HTLV-1-associated bronchiectasis affects individuals of diverse genetic backgrounds infected with HTLV-1 strains other than HTLV-1c. Although HTLV-1 associated pulmonary disease in Japan is thought to be largely sub-clinical[14], published clinical details are limited and prospective survival studies have not been performed.

Consistent with other HTLV-1 associated inflammatory diseases[1,14], the median baseline HTLV-1c pVL of subjects with airways inflammation was substantially higher than that of asymptomatic subjects. For example, the median HTLV-1c pVL in subjects with HAM/TSP is between 7-fold and 16-fold greater than that of asymptomatic subjects[22][28][30–32]. Among subjects with HAM/TSP, higher HTLV-1 pVL correlates with more severe motor weakness[31] and more rapid neurological progression[32]. We previously demonstrated that HTLV-1-infected Indigenous adults have more diffuse bronchiectasis[5] and that a higher HTLV-1c pVL correlates with more extensive pulmonary injury[6]. In contrast to HTLV-1-mediated inflammation in other tissues, pulmonary parenchymal injury can result in directly life-threatening complications, including respiratory failure[5]. Among subjects with airways disease in whom HTLV-1 oligoclonality could be studied, there was no difference in the median OCI when compared to that of asymptomatic subjects. This suggests that higher HTLV-1c pVL were due to an increased number of infected clones rather than clonal expansion, which is consistent with the conclusion previously reported for subjects with HAM/TSP[20]

Increased mortality due to a specific HTLV-1-associated inflammatory disease has not been prospectively demonstrated previously. However, an excess mortality that is not attributable to ATL or currently recognized HTLV-1-associated inflammatory diseases has been reported in other endemic areas. Adjusted hazard ratios of death are 1.3[10] to 1.77–1.87[9] in Japanese outpatient cohorts, and 3.8 and 2.3 for young and middle-aged adults, respectively, in a community-based cohort in Guinea-Bissau[11,12]. In a study that included only 48 HTLV-1-infected subjects in Guinea-Bissau, mortality was associated with higher HTLV-1 pVL[16]. In Japan, excess mortality was attributed to non-neoplastic diseases, most commonly unspecified kidney and cardiac conditions[9,10]. Although the ASH cohort included subjects with established ESKD and heart disease, HTLV-1 infection was only associated with bronchiectasis-related deaths, and this effect was only revealed after stratifying by HTLV-1c pVL. The difference between studies in the clinical conditions associated with excess mortality may reflect the high burden of illness in our hospital-based cohort, the inability to control for comorbid conditions in other studies, and differences in the social circumstances of the various study populations.

The strengths of this prospective cohort study include the recruitment of nearly all eligible subjects with a discharge diagnosis of bronchiectasis, the use of cHRCT for diagnosis and the blinding of ASH researchers to the HTLV-1 serostatus of subjects and of those who performed HTLV-1 studies to their clinical state. Nevertheless, some design limitations must be recognized. First, investigations to exclude other causes of airways disease could not be performed in all cases. However, consistent with previous studies[5,6], a specific aetiology was rarely found among >70% of subjects who were screened for conditions generally associated with bronchiectasis[17]. Although an effect of childhood respiratory infections cannot be excluded in the present study, we previously found HTLV-1 infection to be the major predictor of adult bronchiectasis in a case-control study that controlled for such infections[6]. Second, only subjects with a discharge diagnosis of bronchiectasis were specifically targeted for recruitment. Twelve subjects with COPD (HTLV-1 infected, 4; HTLV-1 uninfected, 8) were incidentally recruited but not examined by cHRCT because they did not clinically warrant further imaging[17]. The contribution of HTLV-1c infection to less severe respiratory disease than that associated with a discharge diagnosis of bronchiectasis, and the validity of our conclusions in a community setting, require further study. Finally, the absence of an association between strongyloides seropositivity and higher HTLV-1c pVL may be due to the fact that strongyloides serology was assayed in subjects without symptomatic strongyloidiasis.

In summary, HTLV-1c infection and higher HTLV-1c pVL were strongly linked to airways inflammation in a hospital-based cohort of Indigenous Australian adults. Furthermore, higher baseline HTLV-1c pVL prospectively predicted death due to bronchiectasis, which may result from more extensive disease[5,6], predisposing to life-threatening complications[5] among subjects who are unable to control HTLV-1 replication[6]. Elucidating the causes of higher mortality among people infected with HTLV-1 is relevant to an estimated 20 million people living with HTLV-1 infection in resource-poor areas[2] where the impact of HTLV-1 infection has been little studied.

## Supporting information

S1 Checklist Strobe checklist. (DOC)
